# Ventricular fibrillation in patients with pathogenic filamin C variants: Even a possibility with normal left ventricular ejection fraction and absent late gadolinium enhancement

**DOI:** 10.1016/j.hrcr.2024.11.010

**Published:** 2024-11-19

**Authors:** Stephan A.C. Schoonvelde, Peter-Paul Zwetsloot, Sing-Chien Yap, Alexander Hirsch, Marjon A. van Slegtenhorst, Judith M.A. Verhagen, Michelle Michels

**Affiliations:** 1 Department of Cardiology, Thorax Center, Cardiovascular Institute, Erasmus Medical Center, Rotterdam, The Netherlands; 2 Netherlands Heart Institute, Utrecht, The Netherlands; 3 Department of Radiology and Nuclear Medicine, Erasmus Medical Center, Rotterdam, The Netherlands; 4 Department of Clinical Genetics, Erasmus Medical Center, Rotterdam, The Netherlands

**Keywords:** Filamin C, Arrhythmogenic cardiomyopathy, Ventricular fibrillation, Sudden cardiac death, Late gadolinium enhancement

**Key Teaching Points**

• Filamin C truncating variant (*FLNCtv*) cardiomyopathies are characterized by heart failure and malignant ventricular arrhythmias. Current guidelines recommend implantable cardioverter-defibrillators for patients with *FLNC*tv cardiomyopathies who have a left ventricular ejection fraction <45% or evidence of late gadolinium enhancement.
• This case describes a patient with *FLNC*tv cardiomyopathy who experienced ventricular fibrillation despite the absence of these traditional risk factors. Magnetic resonance imaging revealed biventricular dilation with preserved biventricular function and no late gadolinium enhancement.
• Inherited cardiomyopathies may have mechanisms for ventricular arrhythmias that are not yet fully understood. Further research is needed to elaborate these mechanisms and improve risk stratification in these patients. A gene-specific approach may be appropriate.

## Introduction

Filamin C (FLNC) is a structural protein that is expressed in striated muscle, providing structural stability to the sarcomere.^1^ Pathogenic variants in the *FLNC* gene have been associated with various disease phenotypes.^2^ The genotype–phenotype correlation from pathogenic *FLNC* variants is modulated by the location of the variant within the *FLNC* gene, and variants associated with 1 type of myopathy or cardiomyopathy are not predicted to be able to cause another phenotype.^2^ Truncating *FLNC* variants (*FLNC*tv) are predicted to cause dilated or arrhythmogenic cardiomyopathy through haploinsufficiency via nonsense-mediated messenger RNA decay, which causes weakened structural adhesion. In comparison, missense variants are associated with hypertrophic or restrictive cardiomyopathies. Myofibrillar myopathies, however, are mostly caused by missense variants or (more rarely) truncating variants residing in specific immunoglobulin-like domains of the *FLNC* gene. *FLNC*tv-related cardiomyopathies are associated with heart failure with reduced ejection fraction and ventricular arrhythmias (VAs) and pose a considerable risk of sudden cardiac death (SCD).^3^^,^^4^ VAs may originate from regions of myocardial ischemia, the Purkinje fibers, the ventricular outflow tracts, or from areas of fibrosis, but its precise substrate is currently not known for *FLNC*tv.^5^ Pathogenic *FLNC* variants are considered a risk factor for VA and, according to current guidelines, an implantable cardioverter-defibrillator (ICD) implantation is indicated when diagnosed in combination with a left ventricular (LV) ejection fraction of <45% and late gadolinium enhancement (LGE).^6^^,^^7^

We describe the case of a patient who presented with an out-of-hospital cardiac arrest due to ventricular fibrillation and in whom a known pathogenic *FLNC*tv was found. Cardiovascular magnetic resonance (CMR) imaging showed biventricular dilation, but with a biventricular normal ejection fraction and notably without the presence of any LGE. This case supports the arrhythmogenicity of *FLNC*tv; however, the essential factors for primary prevention ICD implantation (ie, a decreased LV function and the detection of LGE) are absent, suggesting its arrhythmogenicity might be dependent on other factors as well.^6^^,^^7^

## Case report

**Figure 1:**
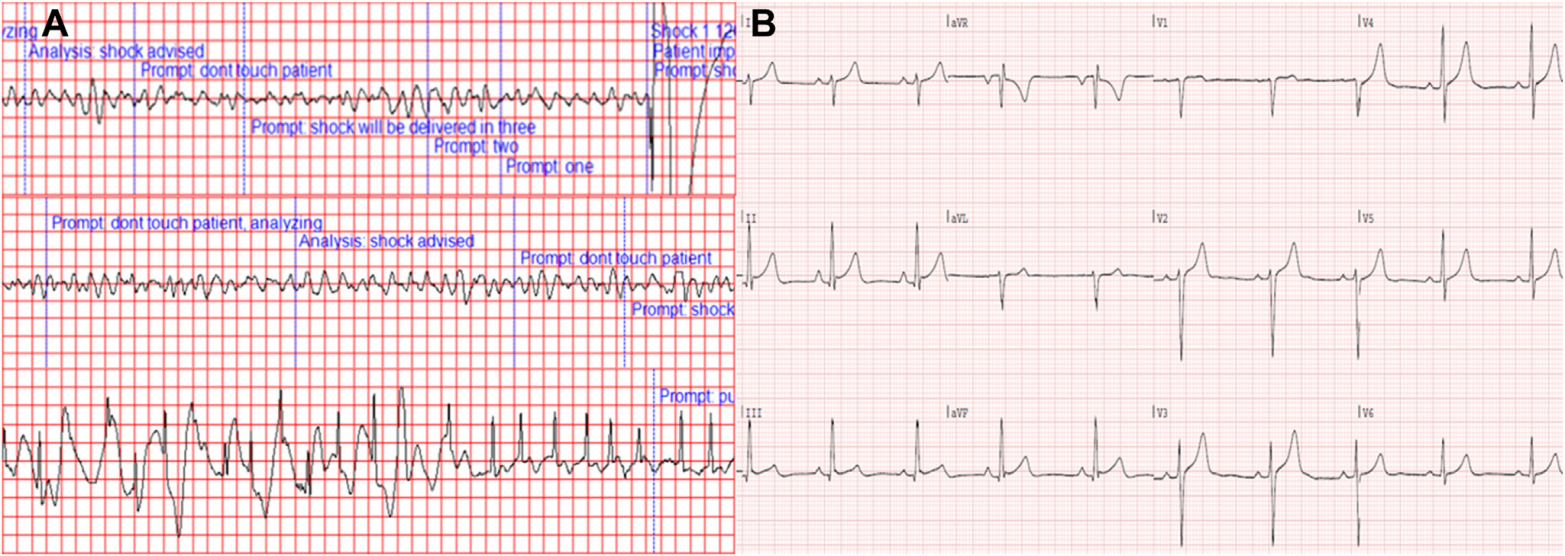
**A:** Tracings from the automatic external defibrillator showing 2 instances of appropriate shock therapy application during episodes of ventricular fibrillation. The last tracing shows the cessation of cardiopulmonary resuscitation on return of spontaneous circulation with sinus rhythm. **B:** Electrocardiogram taken shortly after stabilization showing a sinus rhythm of 56 beats/min, right axial deviation, normal PR, QRS, and QT times and normal repolarizations. There are J waves in the inferior leads with ST-segment elevations of <1 mm. The QTc dispersion was calculated to be 38 milliseconds.

An 18-year-old man with an unremarkable medical history presented with witnessed out-of-hospital cardiac arrest at work moments after using the toilet. According to bystanders, he appeared pale and diaphoretic just before losing consciousness. Basic Life Support was initiated without delay and ventricular fibrillation (Figure 1A) was converted to sinus rhythm after 4 applications of automatic external defibrillator therapy. The patient arrived at the hospital sedated with spontaneous circulation. Initial 12-lead electrocardiogram showed ST-elevation consistent with post-resuscitation ischemia, which resolved on stabilization (Figure 1B). Bedside transthoracic echocardiography ruled out acute pathology, such as cardiac tamponade. Computed tomography angiography excluded acute intracranial pathology and pulmonary embolism. Laboratory testing revealed normal electrolytes and normal thyroid function. Urinary toxicology screening was negative for amphetamines, barbiturates, cannabis, cocaine, and opiates. Other possible etiologies for cardiac arrest, such as hypothermia, hypoxia, and hypovolemia were ruled out, and the patient was admitted to the intensive care unit for targeted temperature management. After recovery from intensive care, the patient described a negative family history for cardiac disease or SCD, and reported the use of recreational 3,4-methylenedioxymethamphetamine (ie, ecstasy) 5 days prior to the event; frequent use of creatine as a sporting supplement; and recreational cannabis consumption. He reported engaging in recreational strength training but does not participate in competitive or endurance sports.

**Figure 2:**
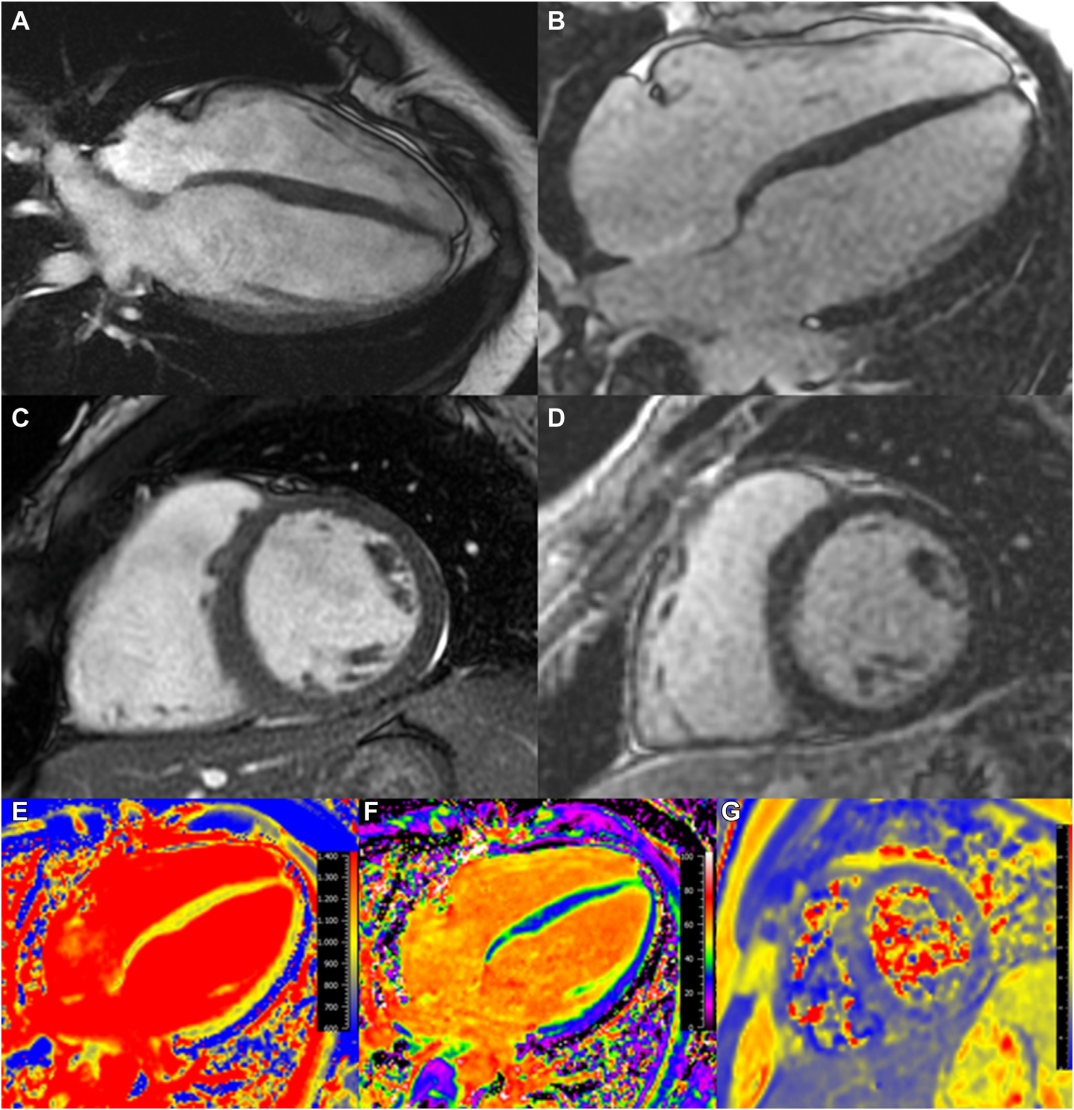
Cardiovascular magnetic resonance imaging. End-diastolic steady-state free precession cine images from the (**A**) 4-chamber and (**C**) mid-ventricular short-axis views. **B, D:** Corresponding late gadolinium enhancement (LGE) images. Note the lack of LGE. **E, F:** T1 mapping with normal T1 and extracellular volume (ECV) values (basal septal native T1 value of 978 milliseconds with an ECV of 30.9%, both within normal limits for our scanner (Signa Artist 1.5T, GE Healthcare).^8^**G:** T2 mapping of the short-axis myocardium showing normal T2 values (mid-myocardial 50 milliseconds).

Five days after the cardiac arrest, CMR was performed (Figure 2), which revealed a substantially dilated nonhypertrophied LV with normal biventricular systolic function (LV end-diastolic volume of 135 mL/m^2^ and LV ejection fraction of 60%) and dilated right ventricle (right ventricular [RV] end-diastolic volume of 142 mL/m^2^ and ejection fraction of 59%). There was a high LV stroke volume of 170 mL (indexed cardiac output 4.0 L/min/m^2^). T1 mapping showed normal native T1 values and extracellular volume, suggesting no interstitial fibrosis. T2 mapping showed normal T2 values and T2-weighted turbo spin echo images did not show myocardial regions with increased signal intensity suggestive of myocardial edema. There was no LGE. Supplemental cine images are provided (Supplemental Videos 1 and 2).

Clinical workup included a negative ajmaline provocation test and a normal exercise test. Clinical cardiac telemetry revealed sporadic (ie, <1%) premature ventricular contractions, and no significant arrhythmias were detected. A subcutaneous ICD for secondary prevention was implanted. The patient was discharged after a full recovery. No outpatient Holter monitoring was performed, and subsequent ICD monitoring detected no arrhythmias. Outpatient follow-up of 17 months has been uneventful to date. Genetic testing was performing using next-generation sequencing of a targeted arrhythmia gene panel, followed by a virtual exome-based panel of cardiomyopathy-related genes. The arrhythmia panel (Supplemental Material) was negative, returning no pathogenic genetic variants. However, the cardiomyopathy panel (Supplemental Material) revealed the truncating c.6864_6867dup, p.(Val2290Argfs∗23) variant in the *FLNC* gene (GenBank accession number NM_001458.4). This Dutch founder variant is associated with left-dominant dilated/arrhythmogenic cardiomyopathy with significant phenotypical variability, as described previously by our group.^9^ RV dilation with preserved RV ejection fraction, as seen concomitantly with the LV dilation in this patient, was also observed in that cohort. Cascade screening identified the same genotype in the patient's male sibling, who exhibited normal biventricular function and dimensions with mild subepicardial LGE on CMR, and in the patient's father, who showed normal biventricular function and dimensions without LGE on CMR. Cardiac screening in the mother revealed no cardiac phenotype and the *FLNC* variant was absent.

## Discussion

This report describes a case of aborted SCD in a patient with an *FLNC*tv and a cardiac phenotype of biventricular dilation in the absence of traditional risk factors. Other causes of VA were excluded. *FLNC*tv-related cardiomyopathies are associated with a high burden of arrhythmias, and the presence of a *FLNC*tv in combination with a decreased LV ejection fraction (<45%) and the presence of LGE are considered important risk factors for VA.^3^^,^^4^^,^^6^^,^^7^ The LGE in *FLNC*tv-related cardiomyopathies is classically distributed in a ring-like pattern, which is a known risk factor for VA.^10^ However, the patient in this report presented with ventricular fibrillation without detected LGE, and with biventricular dilation with a preserved LV and RV function. Both T1 and T2 mapping were normal in this patient, suggesting no active inflammatory process or other underlying cardiac pathology.

The latest ESC guidelines on cardiomyopathies introduced a new cardiomyopathy classification, including nondilated left ventricular cardiomyopathy.^6^ However, although this patient does not fulfill any of the current cardiomyopathy definitions, prior classifications have considered isolated ventricular dilation as an early phase of the phenotypical expression.^11^ Earlier research has only implicated LV dilation as a risk factor for sustained VA in the combined presence of a decreased LV ejection fraction.^12^^,^^13^ The presence of biventricular dilation in this case (which was in excess of his level of exercise, not explained by shunting or a fistula, or the consequence of anemia), without decreased function and without detected myocardial fibrosis, illustrates a limitation in the understanding of the pathophysiology of VA in patients with inherited cardiomyopathies. Previous research has found that nonischemic cardiomyopathy patients with ventricular fibrillation often had no LGE.^14^

VA can be caused by different mechanisms, such as abnormal impulse formation or reentry circuits. Large areas of fibrosis can be the substrate for macro-reentry, and this may explain the high risk of SCD in inherited cardiomyopathy patients with extensive LGE. However, this case did not show any LGE on CMR, which may be due to a true absence of fibrosis, or perhaps as a consequence of insufficient sensitivity of current CMR techniques.

## Conclusion

Current cardiomyopathy guidelines suggest using gene-specific risk scores when possible.^6^ Some variants, including *FLNC*tv, are known to have an increased risk of VA, compared with patients with genotype-negative cardiomyopathy. This case illustrates that VA can occur in patients with preserved function and without LGE, and the current knowledge falls short in predicting risks in patients with *FLNC*tv.^15^

Future research is needed to better understand the pathogenesis of VA in patients with inherited cardiomyopathies. Future standardized electrophysiologic research may be warranted to better understand arrhythmia inducibility in such patients. Ultimately, a gene-specific understanding and individualized approach may be required in the management of patients with pathogenic cardiomyopathy-related gene variants.

## Disclosures

Peter-Paul Zwetsloot is partially funded through a Dutch Heart Foundation Public Private Partnership Grant (CARMA, grant 01-003-2022-0358) and has received speaker fees from MedNet and consultancy fees from Bayer, Alnylam, and Bristol Myers Squibb. Sing-Chien Yap has received honoraria from Boston Scientific, Medtronic, Abbott, Biotronik, Acutus Medical, and Sanofi. In addition, he has received institutional research grants from Medtronic, Biotronik, and Boston Scientific. Alexander Hirsch received an institutional research grant and consultancy fees from GE Healthcare and speaker fees from GE Healthcare, Bayer, and Bristol Myers Squibb. He is also a member of the medical advisory board of Medis Medical Imaging Systems and was an MRI Core Lab supervisor of Cardialysis BV until 2022. Michelle Michels received an research grant and speakers fee from Bristol Meyers Squibb, consultancy fees from Cytokinetics, and speakers fee from Pfizer; the rest of the authors have no conflicts of interest.
