# Supplementary material for: Ventricular fibrillation in patients with pathogenic filamin C variants: Even a possibility with normal left ventricular ejection fraction and absent late gadolinium enhancement
Source: HeartRhythm Case Rep. 2024 Nov 19;11(3):198–202. doi: 10.1016/j.hrcr.2024.11.010 (PMC11962988; doi:10.1016/j.hrcr.2024.11.010)
Supplement: Supplemental Materials [file mmc1.docx]

**Supplemental Materials**

**Supplement S1**

**Video S1** – Supplemental cardiovascular magnetic resonance cine image from the four-chamber perspective.

**Supplement S2**

**Video S2** – Supplemental cardiovascular magnetic resonance cine images from the short-axis perspective.

**Supplement S3**

Next-Generation Sequencing Arrhythmia Panel

(Available from: Amsterdam University Medical Centers; <https://genoomdiagnostiek.nl/en/panels/ngs-arrhythmia/>)

**Panel version:** SCDv9;ARTMtypeAv2

Containing genes and haplotypes:

ABCC9, AKAP9, ANK2, CACNA1C, CALM1, CALM2, CALM3, CASQ2, CAV3, DPP6, GJA5, HCN4, HEY2, HOOK3, JPH2, KCNA5, KCND3, KCNE1, KCNE2, KCNH2, KCNJ2, KCNQ1, LAMP2, LMNA, MYL4, NKX2-5, NPPA, PKP2, PLN, PRKAG2, RANGRF, RRAD, RYR2, SCN5A, SLC4A3, SNTA1, TCAP, TECRL, TNNI3K, TNNT2, TRDN, TRPM4.

**Supplement S4**

Whole Exome Sequencing Gene Package Cardiomyopathy

(Available from: Erasmus Medical Center Department of Clinical Genetics; <https://www.erasmusmc.nl/-/media/erasmusmc/pdf/1-themaspecifiek/next-generation-sequencing/cardiomyopathy_info_file_v12_pdf>)

**Panel version:** version 12

Containing genes:

ACAD9, ACTC1, ACTN2, ALPK3, BAG3, BAG5, CDH2, CRYAB, CSRP3, DES, DSC2, DSG2, DSP, DTNA, EMD, EYA4, FHL1, FHOD3, FKTN, FLNC, GATAD1, GLA, HCN4, ILK, JPH2, JUP, KIF20A, LAMP2, LMNA, MIB1, MYBPC3, MYH6, MYH7, MYL2, MYL3, MYLK2, MYOZ1, MYOZ2, MYPN, NEBL, NEXN, NKX2-5, PDLIM3, PKP2, PLN, PPA2, PPCS, PPP1R13L, PRDM16, PRKAG2, PTPN11, RAF1, RBM20, RYR2, SCN5A, SGCD, SOS1, TAFAZZIN, TBX20, TCAP, TMEM43, TNNC1, TNNI3, TNNI3K, TNNT2, TPM1, TRIM63, TTN, TTR, TXNRD2, VCL.
